# A novel prognostic biomarker in progression free survival for patients with cervical cancer, glucose to c-reactive protein ratio (GCR)

**DOI:** 10.1186/s12885-024-12347-x

**Published:** 2024-05-23

**Authors:** Mehmet Emin Buyukbayram, Zekeriya Hannarici, Aykut Turhan, Alperen Akansel Caglar, Pınar Çoban Esdur, Mehmet Bilici, Salim Basol Tekin, Burak Erdemci

**Affiliations:** 1 Department of Medical Oncology, Ataturk University Faculty of Medicine, Erzurum, Turkey; 2 Department of Radiation Oncology, Ataturk University Faculty of Medicine, Erzurum, Turkey

**Keywords:** Cervical cancer, glucose/c-reactivated protein ratio, Progression-free survival

## Abstract

**Background:**

Cervical cancer is a tumor with high morbidity and mortality. The importance of inflammatory and metabolic parameters affecting progression-free survival (PFS) and overall survival (OS) has been investigated more intensively recently. We aimed to investigate the effect of glucose/c-reactive protein (CRP) ratio [GCR], which shows these two parameters together, on PFS in cervical cancer.

**Methods:**

We retrospectively included 90 patients with adenocarcinoma and squamous cell carcinoma of the cervix. The effects of clinical variables, inflammatory and glycemic parameters on PFS and OS were analyzed by Kaplan-Meier method. The data were compared with the healthy control group of 90 individuals using the independent t test. The effect of parameters on mortality was analyzed using ROC curves and cut off values were determined.

**Results:**

Glucose, CRP, CRP/lymphocyte ratio (CLR) and GCR were statistically significant in predicting mortality (*p* < 0.05). Disease stage, glucose, CRP, CLR and GCR were associated with overall survival. CRP, CLR and GCR were associated with progression-free survival (*p* < 0.05). In multivariate analysis, GCR was prognostic for PFS (*p* = 0.025). GCR was statistically significant while compared with the patient and healthy control group (*p* < 0.001).

**Conclusion:**

In cervical cancer, GCR rate was found to be prognostic independent of stage. Higher GCR rate was associated with longer PFS duration.

**Supplementary Information:**

The online version contains supplementary material available at 10.1186/s12885-024-12347-x.

## Introduction

Cervical cancer is the 4th most common cause of cancer death worldwide and the 4th most common cancer of the genital system among malignancies in the female sex [1, 2]. According to The Global Cancer Observatory (GLOBOCAN) 2020 database, there were 604,127 new cases and 341,831 mortalities per year (3). Human papilloma virus (HPV) vaccines and routine screening programs have reduced the incidence and mortality of cervical cancer in developed countries. High incidence and mortality rates persist in undeveloped and developing countries [4–9]. It is an important cause of mortality and morbidity in young and middle-aged women aged 20 to 39 years [10].

Surgery, radiotherapy, chemotherapy and chemoradiotherapy can be performed in the treatment of cervical cancer [11]. Surgery can be performed in the early stage, chemoradiotherapy in the locally advanced stage, and chemotherapy or chemoradiotherapy in the advanced stage [12]. In advanced stages and locally advanced stages, if the disease recurs after primary treatment, chemotherapy options are limited, and life expectancy is shortened. In the United States, the 5-year survival rate for cervical cancer is 57% for locally advanced disease, 16% for stage 4 disease, and 5% for recurrent disease [13, 14]. Factors other than stage that affect progression-free survival (PFS), which is the time from primary treatment of the disease to recurrence, are unknown. The increase in mortality and morbidity reveals the importance of potential prognostic factors for PFS in patients with cervical cancer [15].

Chronic inflammation caused by bacterial, viral or parasitic reasons can induce cancer formation [16]. It has been determined that the immune response occurring in leishmania infestations may have anti-tumor or protumor properties [17]. It has been shown that Bacteroides species play a role in colon cancer, hepatitis B and hepatitis C viruses play a role in hepatocellular cancer, and the inflammatory response and released cytokines play a role in tumor formation and tumor progression [18, 19]. Chronic inflammation caused by the response to infections can dysregulate immunity. This has been shown in human leprosy infections [20]. Chronic inflammatory environment and impaired immune regulation cause tumor formation, tumor progression, and increased ability of the tumor to metastasize. This process is continued by creating the tumor microenvironment through antigen presentation and cytokine release by the tumor. Chronic inflammation of HPV plays an important role in the development of cervical cancer. C reactive protein (CRP), which is a good indicator of inflammation, has been found to be associated with prognosis in cervical cancer as well as in colorectal cancer and esophageal cancer [12, 21].

Inflammatory parameters neutrophil/lymphocyte ratio (NLR), Systemic inflammatory index (SII) and nutritional parameters prognostic nutritional index (PNI) may play a role in tumor prognosis. In a meta-analysis conducted in cervical cancer, it was found that high NLR was associated with low overall survival (OS) and short PFS and indicated advanced stage disease. In studies conducted in esophageal cancer and hepatocellular carcinoma, the correlation of NLR with prognosis has been shown [22–24]. SII was found to be associated with OS in early stage cervical cancer [25]. PNI has been found to be associated with prognosis in many studies, while it has not been found to be prognostic in some studies [26]. Studies on inflammatory indices and nutritional markers in many tumor types are ongoing.

A relationship between fasting glucose and gynecological malignancies has been demonstrated [27]. Hyperglycemia affects tumor response to treatment, tumor recurrence, and survival prognosis. Hyperglycemia has a poor prognostic effect by causing excessive glucose utilisation and tumor growth, genetic damage with free oxygen radicals, increase in insulin and growth factors, and increase in inflammatory cytokines [28]. Glucose metabolism in cancer development, glycemic index and glycemic load parameters have been found to be related with colorectal cancer and endometrial cancer. It has been shown in some studies that fasting glucose is related with prognosis in cervical cancer [29].

The parameters that predict stage-independent PFS in cervical cancer are limited and life expectancy is shorter if cervical cancer progresses. The correlation between glucose and CRP, both metabolic and inflammation indicators, and cervical cancer prognosis has not been reported in the literature. Therefore, we aimed to investigate the prognostic significance of glucose/c-reactivated protein ratio (GCR), which is both a metabolic and inflammation indicator and a relatively new marker.

## Materials and methods

We included 90 patients with stage 1A1-4B squamous cell carcinoma and adenocarcinoma of the cervix between January 2009 and December 2021. Patients with incomplete file data, chronic inflammatory diseases, and other known malignancies were excluded. Demographic data such as age, Eastern Cooperative Oncology Group (ECOG) performance score; histological type, staging and treatments were obtained from patient files. 90 healthy individuals were enrolled as healthy control group. Those without known cancer and chronic inflammatory disease were included in the healthy control group.

Leukocyte, lymphocyte, neutrophil, hemoglobin, platelet, glucose, CRP, albumin and carcinoembryonic antigen (CEA) values were obtained from the hospital information system. NLR is the ratio of neutrophil count to lymphocyte count. SII was calculated as platelet (P) × neutrophil (N)/lymphocyte (L) and PNI was calculated as (10× albumin (g/L) + (0.005 × total lymphocyte count). Ethics committee approval was obtained from Atatürk University Faculty of Medicine ethics committee (B.30.2.ATA.0.01.00/601 − 146). All procedures were in accordance with the Declaration of Helsinki.

### Statistical analysis

Overall survival (OS) was calculated from the date of diagnosis of cervical cancer to the date of death or last follow-up. For PFS, the dates of disease recurrence, progression or death from the date of diagnosis were considered as progression. Statistical analyses were performed using “IBM SPSS Statistics for Windows. Version 25.0 (Statistical Package for the Social Sciences, IBM Corp. Armonk, NY, USA)”.

Descriptive statistics are presented as n and % for categorical variables and Mean ± SD for continuous variables. Significant results in GCR and CLR were compared with the healthy control group using the independent t test. The results of ROC Curve analysis for the prediction of mortality by various numerical parameter scores are given. Kaplan-Meier method was used to compare OS and PFS durations between various clinical parameter groups. Multivariate cox regression results of various clinical factors on the risk of death and PFS are given and *p* < 0.05 is considered statistically significant.

## Results

Our study included 90 patients and the mean age was 52 (30–76) years. The number of patients with ECOG performance scores of 0, 1 and 2 were 18, 37 and 3, respectively. There were 78 (87.6%) patients with squamous cell carcinoma histology and 11 (12.4%) patients with adenocarcinoma histology. The number of stage 1–3 patients was 58 (69.0%) and stage 4 patients was 26 (31.0%). During follow-up, progression was observed in 35 (41.2%) patients and exitus in 22 (24.4%) patients. Table 1 shows the distribution of demographic and clinical information of the patients. The control group consisted of 90 female patients and median age 50 (30–76).

**Table 1:** Data on demographic and clinical characteristics

	*N*	%
ECOG		
0	18	31,0
1	37	63,8
2	3	5,2
Pathological type		
Squamous	78	87,6
Adenoca	11	12,4
Stage		
1–3	58	69,0
4	26	31,0
Lymph Node		
No	26	32,9
Pelvic	40	50,6
Paraaortic	13	16,5
CRT		
No	12	14,6
Yes	70	85,4
CRT regimen		
Cisplatin	61	93,8
Other	4	6,2
Metastatic CRT regimen		
Cisplatin + paclitaxel	2	6,7
Carboplatin + paclitaksel	7	23,3
Paclitaxel + Carboplatin + Bevacizumab	5	16,7
Cisplatin + paclitaxel + Bevacizumab	10	33,3
Cisplatin + Gemcitabine + Bevacizumab	2	6,7
Paclitaxel + Topotecan + Bevacizumab	1	3,3
Cisplatin + Capecitabine	1	3,3
Other	2	6,7
Progression		
No	50	58,8
Yes	35	41,2
Mortality		
Live	68	75,6
Exitus	22	24,4
Mean follow-up time	68,14 ± 55,13	
Mean age	52,28 ± 11,91	

CRT, chemoradiotherapy; ECOG, Eastern Cooperative Oncology Group

Cut-off values were calculated using the Roc curve to determine the presence of mortality. The prediction of GCR (*p* = 0.001), CLR (*p* = 0.006), CRP (*p* = 0.001) and glucose (*p* = 0.048) parameters were found statistically significant. In the ROC analysis for GCR, the AUC was 0.778 (95% [CI], 0.658–0.889). Cut off ≤ 0.42, GCR values have a sensitivity of 69.2% and a specificity of 64.4%. In the ROC analysis for CLR, the AUC is 0.755 (95% [CI], 0.614–0.895). Cut off ≥ 5.17 CLR values have a sensitivity of 66.7% and a specificity of 66.7%. In the ROC analysis for CRP, the AUC is 0.792 (95% [CI], 0.655–0.918). Cut off ≥ 12.59 CRP values have a sensitivity of 66.7% and a specificity of 66.7%. In the ROC analysis for glucose, the AUC was 0.657 (95% [CI], 0.511–0.802). Cut off ≥ 5.79 glucose values have a sensitivity of 66.7% and a specificity of 64.6%.

However, the prediction of NLR, SII, hemoglobin, CEA and PNI parameters were not statistically significant (*p* > 0.05). Table 2 shows the analysis of the predictive values of these parameters for mortality.

**Table 2:** Analysis of the predictive value of various parameter values in discriminating mortality

Variables	AUC	%95 CI	Cut-off	Sensitivity (%)	Specificity (%)	*p*
NLR	0.596	0.426–0.766	≥ 2.87	53,3	56,3	0.298
SII	0.629	0.457–0.802	≥ 890.54	66,7	64,6	0.163
Glucose	0.657	0.511–0.802	≥ 5.79	66,7	64,6	**0.048**
CRP	0.792	0.655–0.918	≥ 12.59	66,7	66,7	**0.001**
Hemoglobin	0.473	0.283–0.662	≥ 12.25	53,3	50,0	0.766
CEA	0.497	0.315–0.679	≥ 1.75	46,7	47,9	0.976
GCR	0.778	0.658–0.889	≤ 0,42	69,2	64,4	**0.001**
CLR	0.755	0.614–0.895	≥ 5.17	66,7	66,7	**0.006**
PNI	0.569	0.355–0.783	≤ 40,80	53,8	53,3	0.450

**Fig. 1:**
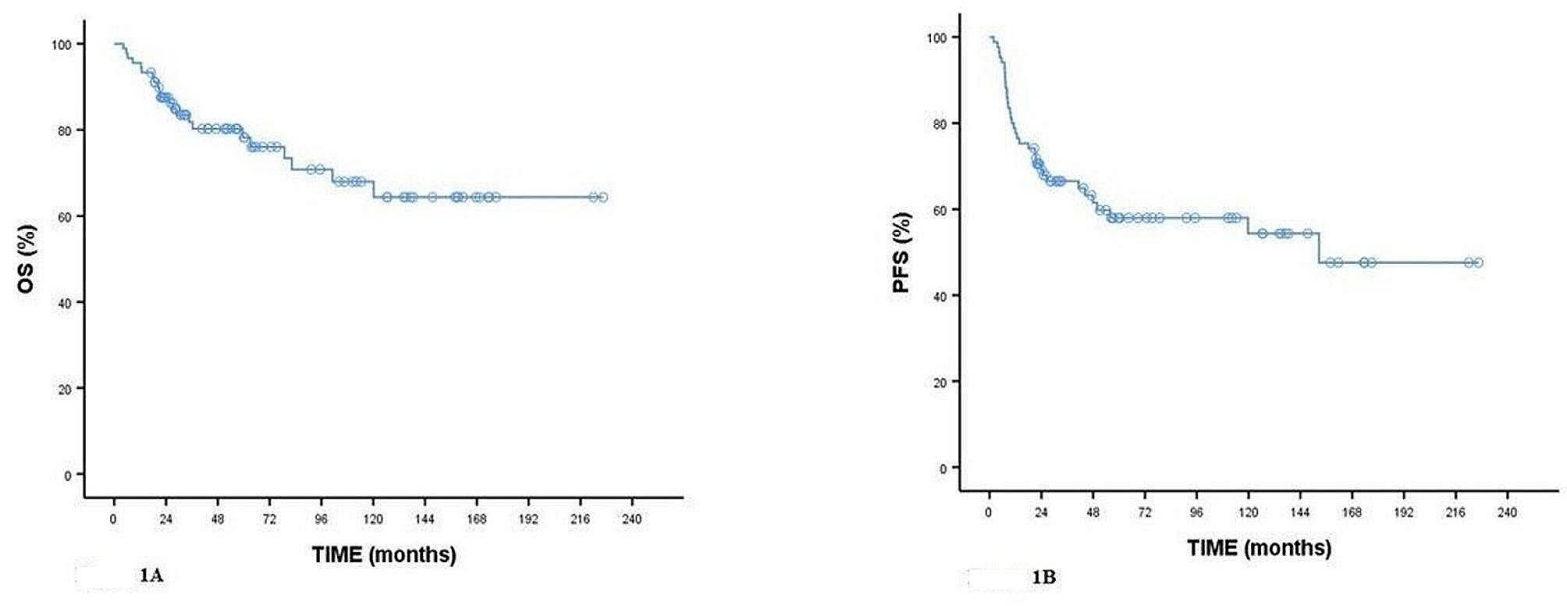
Kaplan-Meier curves for overall survival in cervical cancer (A) and progression-free survival in cervical cancer (B)

**Fig. 2:**
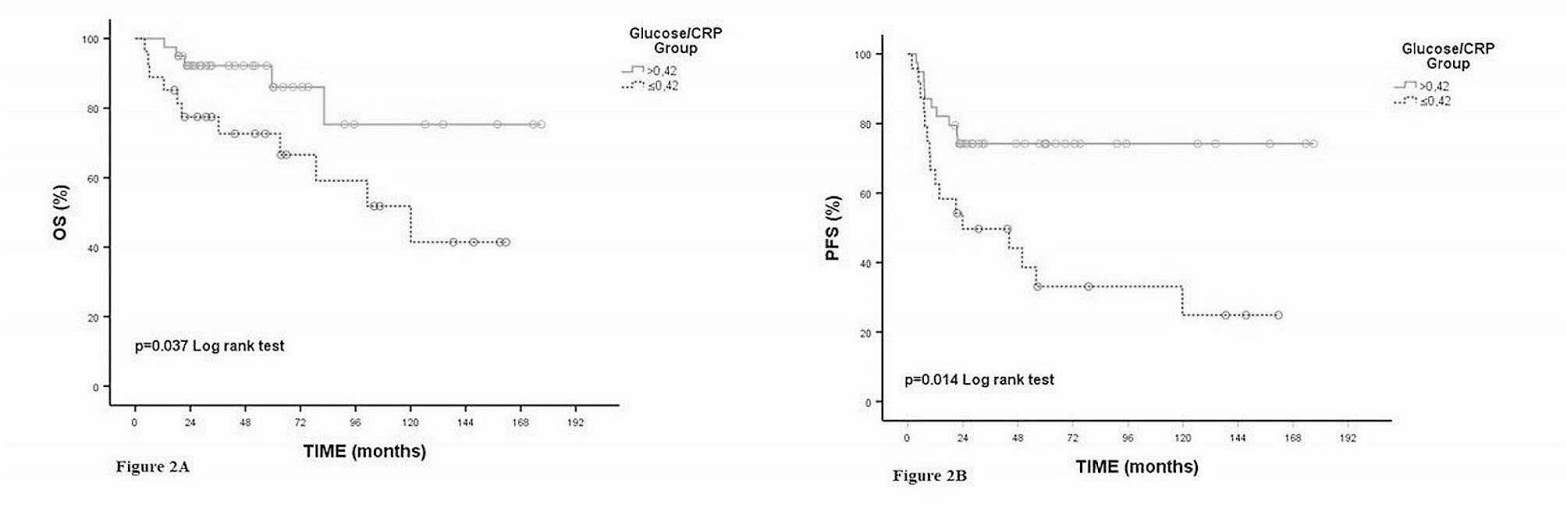
Kaplan-Meier curves for overall survival in cervical cancer (A) and progression-free survival in cervical cancerrelative to glucose/CRP ratio (B)

AUC, area under curve ; %95CI, confidence interval; CLR, CRP/lymphosite ratio; CRP, C reactive protein; GCR, glucose/CRP ratio NLR, neutrophil/lymphocyte ratio; PNI, prognostic nutritional index; SII, systemic inflammayory index; CEA, carcinoembryonic antigen;

Overall median OS (months) was not reached in patients (Fig. 1A). Median OS (months) according to the stage groups was statistically significant (*p* = 0.045). Median OS (months) was not reached in Stages 1–3 and Stage 4. Median OS (months) according to glucose groups was statistically significant (*p* = 0.034). Median OS (months) was not reached in the group with a glucose value < 5.79. In the group with glucose value ≥ 5.79, median OS (months) were 120.16 (95%CI: 63.84-176.49). Median OS (months) according to CRP groups was statistically significant (*p* = 0.006). Median OS (months) was not reached in the group with CRP value < 12.59. In the group with CRP value ≥ 12.59, median OS (months) were 101.13 (95%CI: 49.01-153.25). Median OS (months) according to GCR groups was statistically significant (*p* = 0.037). Median OS (months) was not reached in the group with GCR value > 0.42. In the group with GCR value ≤ 0.42, median OS (months) was 120.16 (95%CI: 59.06-181.26) (Fig. 2A). Median OS (months) according to CLR groups was statistically significant (*p* = 0.043). OS (months) was not reached in the group with CLR value < 5.17. In the group with CLR value ≥ 5.17, median OS (months) was 120.16 (95%CI: 61.79-178.53).

Overall median PFS (months) was 152.60 (95%CI:-) (Fig. 1B). Median PFS (months) according to stage groups was not statistically significant (*p* = 0.076). Median PFS (months) according to glucose groups was not statistically significant (*p* = 0.131). Median PFS times (months) according to CRP groups were statistically significant (*p* = 0.014). Median PFS (months) was not reached in the group with a glucose value < 12.59. In the group with CRP value ≥ 12.59, median PFS (months) were 44.23 (95%CI: 0.00-93.58). Median PFS (months) according to GCR groups was statistically significant (*p* = 0.004). Median PFS (months) was not reached in the group with GCR value > 0.42. In the group with GCR value ≤ 0.42, median PFS (months) was 23.90 (95%CI: 0.00-67.16) (Fig. 2B). Median PFS (months) according to CLR groups was statistically significant (*p* = 0.031). Median PFS (months) was not reached in the group with CLR value < 5.17. In the group with CRP value ≥ 5.17, median PFS (months) was 49.86 (95%CI: 1.19–98.53) (Table 3).

**Table 3:** Overall survival and progression free survival comparisons of patients

	OS (months)		PFS (months)	
Variables	Median (%95 CI)	*p*	Median (%95 CI)	*p*
Overall	(-)		152,60 (-)	
Stage				
1–3	- (-)	0.045	- (-)	0.076
4	- (-)		49,86 (-)	
Glucose				
< 5,79	- (-)	0.034	- (-)	0.131
≥ 5,79	120,16 (63,84–176,49)		119,73 (0,00-264,97)	
CRP				
< 12,59	- (-)	0.006	- (-)	0.014
≥ 12,59	101,13 (49,01-153,25)		44,23 (0,00–93,35)	
GCR				
> 0,42	- (-)	0.037	- (-)	0.004
≤ 0,42	120,16 (59,06-181,26)		23,90 (0,00–67,16)	
CLR				
< 5,17	- (-)	0.043	- (-)	0.031
≥ 5,17	120,16 (59,06-181,26)		49,86 (1,19–98,53)	

Kaplan Meier curve, Long rank test, *p* < 0.05 statistically significant; CRP, c reactive protein; GCR, glucose/CRP ratio; CLR, CRP/lymphosite ratio; OS, overall survival; PFS, progression free survival

As a result of univariate analyses, stage, glucose, CRP, GCR and CLR variables were found statistically significant in terms of mortality risk (*p* < 0.05) (Table 3). These variables found to be significant in univariate analyses were included in the multivariate cox regression model. According to the results of the multivariate cox regression model, the variables did not show statistical significance on the risk of mortality (*p* > 0.05) (Table 4).

**Table 4:** Multivariate Cox Regression Results for Various Clinical Variables

OS	Multivariate		
Variables	HR (95%CI)		*p*
**Stage (Ref: 1–3)**	2.55 (0.78-8,37)		0.187
**Glucose (Ref:<5,79)**	0.90 (0,26 − 3,07)		0.063
**GCR (Ref:>0,42)**	2.33 (0,68 − 7,96)		0.381
**CLR (Ref:<5,17)**	2.83 (0.84-9,49)		0.860
	*p* = 0.030; -2 Log Likelihood = 103,885		
**PFS**	**Multivariate**		
**Variables**	**HR (95%CI)**	**p**	
**Stage (Ref: 1–3)**	2.14 (0.84-5,48)	0.111	
**Glucose (Ref:<5,79)**	1.98 (0,87 − 4,52)	0.103	
**GCR (Ref:>0,42)**	6.50 (1,26–33,56)	**0.025**	
**CLR (Ref:<5,17)**	0.35 (0.06-1,86)	0.222	
	*p* = 0.009; -2 Log Likelihood = 182,42		

CLR, CRP/lymphosite ratio; CRP, c reactive protein; GCR, glucose/CRP ratio; OS, overall survival; PFS, progression free survival; Ref, reference

As a result of univariate analyses, CRP, GCR and CLR variables were found statistically significant in terms of PFS risk (*p* < 0.05) (Table 3). Stage and glucose variables were included in the multivariate model according to the *p* < 0.200 rule. These variables, which were found significant as a result of univariate analyses, were included in the multivariate cox regression model. According to the results of the multivariate cox regression model, a GCR value ≤ 0.42 (HR: 6.50; 95%CI: 1.26–33.56; *p* = 0.025) increased the duration of PFS (*p* = 0.009, -2 log-likelihood = 182.42) (Table 4). In the Spearman correlation test of GCR with CLR (*r*=-0.950, *p* < 0.001), and CRP (*r*=-0.989, *p* < 0.001) showed a very strong negative correlation (Table 5). CRP (*p* < 0.001), GCR, CLR values showed significant differences between the patient and control groups with independen t test (Table 6). While CRP and CLR values are higher in the patient group, Glucose/CRP values are higher in the control group.

**Table 5:** Spearman correlation test of GCR with CLR and CRP

		GCR	CLR	CRP
GCR	r	1		
	p			
CLR	r	-,950^**^	1	
	p	**< 0,001**		
CRP	r	-,989^**^	,962^**^	1
	p	**< 0,001**	**< 0,001**	

CLR, CRP/lymphosite ratio; CRP, c reactive protein; GCR, glucose/CRP ratio

**Table 6:** Comparison of GCR and CLR with the control group

	Patient Mean ± SD	Control Mean ± SD	*p*
CRP	31,98 ± 54,12	4,26 ± 5,33	< 0.001
GCR	2,42 ± 4,96	30,05 ± 113,90	< 0.001
CLR	24,64 ± 48,59	1,72 ± 2,02	< 0.001

Independent t test

CLR, CRP/lymphosite ratio; CRP, C reactive protein; GCR, glucose/CRP ratio

## Discussion

Although there have been advances in the diagnosis and prevention of cervical cancer, outcomes are poor in advanced and recurrent disease [30]. Studies on parameters affecting disease recurrence and PFS are limited and biomarkers that will predict PFS are needed. In many cancer types, inflammatory parameters (NLR, SII, PNI) can be used in prognostic markers such as PFS and OS, treatment response, tumor diameter and lymph node involvement [31].

Tumors gain their angiogenesis, proliferation, invasion, and metastasis abilities in the inflammatory environment. Locally produced pro-inflammatory and anti-inflammatory cytokines create an inflammatory environment depending on neutrophil activity and lymphocyte response. Some substances, such as VEGF, mitogens, and adhesive glycoproteins secreted from platelets also contribute to this condition [32]. It is known that the environment formed in the tumor microenvironment affects the number of neutrophils, lymphocytes, platelets and the differences in their ratios in peripheral blood [31].

In studies performed with NLR, it was found to be related with survival in cervical cancer. Different results were obtained in studies performed with another index, SII, due to the difference in patient groups. In our study, no significant correlation was found between NLR, PNI and SII and PFS and OS. In their study Huang QT et al. found NLR to be positively related to tumor diameter, correlated with lymph node metastasis, and significant in advanced stage disease unlike our study. In their study Huang H et al. found SII to be significant for OS in cervical cancer that underwent radical resection unlike our study [22–25]. In the study conducted by Jiang et al. with early stage 1–2 A, PNI value was not found significant for PFS and OS similar to our study [26]. The patient group of our study was heterogeneous. The results of our study may be different from some studies in the literature because it included patients at all stages, there were patients with and without surgery, the ethnic origins of the patients may be different, and the cut-off values for NLR and SII.

Hemoglobin was not found to be significant for PFS and OS in our study. In the study conducted by Jiang et al. in stage 1–2 A cervical cancer patients, hemoglobin values were not found significant for PFS and OS similar to our study [26]. Grover et al. found that hemoglobin values were associated with PFS and OS in a study conducted with 1017 patients with stage 1–4 cervical cancer [33]. In the study of Grover et al. hemoglobin value was found to be significant for PFS and OS unlike our study. The reason why may be that our patient population was small, and the number of early-stage patients was high.

CEA was not found prognostic in our study. In the study conducted by Chmura et al., CEA value was found to be low prognostic in early-stage cervical squamous cell carcinoma, while CEA value was found to be prognostic in advanced stage cervical squamous cell carcinoma [34]. In a study conducted by Huang G. et al. in early-stage cervical adenocarcinoma patients, CEA value was found to be prognostic [35]. The reason why CEA was not found to be significant in our study may be that cervical adenocarcinoma and squamous cell carcinoma patients were found together unlike the study of Chmura et al. and the stages of the patients included early and advanced stages together unlike the study of Huang et al.

It is known that advanced stage cervical cancer has a high mortality rate. In our study, the stage of the patient was found to be significant for OS in univariate analysis. In the study conducted by Grover et al. the stage of the disease was found to be significant for OS. There were shorter OS periods in stage 4 compared to stages 1–3 [33]. Our study results were similar.

Cancer cells uptake more glucose than benign cells and the glycolysis system is more active. This positive feedback leads to overutilisation of glucose and increases the produced lactate dehydrogenase and Hypoxia induced factor 1, which in turn affects tumor angiogenesis and metastasis. Increased glucose utilisation causes impaired fasting blood glucose values and has been found to be associated with prognosis in cervical cancer studies [36].

In our study, no statistically significant correlation was found between fasting glucose value and PFS, but it was found to be significantly associated with OS. Lee et al. found high fasting glucose (cut off > 102) to be associated with poor OS in their study in stage 2–4 cervical cancer [37]. Our study results were similar for OS. Ahn et al. found that high fasting glucose levels were associated with lower PFS in early-stage cervical cancer unlike our study [36]. The contrasting situation in PFS may be due to the difference in our patient groups and the difference in cut-off values.

CRP, which is considered as a sensitive marker of systemic inflammatory response, is synthesized from liver cells in response to many pro-inflammatory cytokines. High CRP values were found to be associated with shorter PFS periods and shorter OS in our study. Chmura et al. found that CRP values were associated with prognosis in resectable cervical cancer. Wang et al. found high CRP values as independent risk factors for PFS and OS in early-stage cervical cancer. Polterauer et al. found CRP > 5 to be associated with shorter PFS and OS in their study in stage 1–4 cervical cancer [34, 38, 39]. Our study results were similar.

In our study high CLR rates were associated with shorter OS and shorter PFS. Univariate analysis was significant for PFS and OS. CLR was not significant in multivariate analysis. Okugawa et al. found that pretreatment lymphocyte/CRP ratio was significant for PFS and OS in rectal cancer [40]. Although CLR rate has not been investigated in cervical cancer before, CLR rate was investigated as a new prognostic marker in our study. Further studies in larger patient groups and specific stage groups are needed.

In our study, GCR was found to be significant for OS in cervical cancer in univariate but not in multivariate analysis. However, GCR was shown as a new parameter especially in terms of PFS. GCR was found significant for PFS in both univariate and multivariate analyses (*p* = 0.025). GCR was statistically significant when compared with the patient and healthy control group (*p* < 0.001). In the study conducted by Ruan et al., high values in c-reactive protein triglyceride glucose index (CTI) were found to be associated with poor prognosis in various cancer groups and at different stages [41]. Since this index includes relatively more complex parameters and is not routinely analyzed in cancer patients, it may not be possible to evaluate it for every patient. In this respect, GCR was studied in our study as a slightly simpler parameter. Although there have been previous studies with glucose and CRP, for the first time we demonstrated the prognostic importance of GCR for PFS in cervical cancer and our comparison with the healthy control group was more significant.

Since our study was retrospective, it has some limitations such as the absence of cervical tumors with rare pathologies such as Glassy cell carcinomas, adenosquamous carcinomas, neuroendocrine carcinomas, and the fact that it was a single-center study. We believe that our results should be confirmed by further studies with a multicenter and larger patient sample.

## Conclusion

This is the first study showing the association of GCR with PFS and OS in cervical cancer. Considering that survival rates decrease significantly in recurrent disease, it is important that GCR, which includes the inflammatory and glycemic parameter effective in disease pathogenesis and prognosis, is statistically significant. GCR is affordable and accessible with a simple peripheral blood test increases its value. With further supporting studies, it will be possible to strengthen the clinician’s hand in predicting recurrence in cervical cancer.

### Electronic supplementary material

Below is the link to the electronic supplementary material.

Supplementary Material 1

## Data Availability

The data underlying this article will be shared on reasonable request to the corresponding author.
