# Supplementary material for: A novel prognostic biomarker in progression free survival for patients with cervical cancer, glucose to c-reactive protein ratio (GCR)
Source: BMC Cancer. 2024 May 23;24:626. doi: 10.1186/s12885-024-12347-x (PMC11112963; doi:10.1186/s12885-024-12347-x)
Supplement: Supplementary file 1 — Supplementary Material 1 [file 12885_2024_12347_MOESM1_ESM.docx]

**Figure 1.** Kaplan–Meier curves for overall survival in cervical cancer (A) and progression-free survival in cervical cancer (B)

**Figure 2.** Kaplan–Meier curves for progression-free survival in cervical cancer (A) and overall survival in cervical cancer relative to glucose/CRP ratio (B).
